# Analysis of differences in intraocular pressure evaluation performed with contact and non-contact devices

**DOI:** 10.1186/s12886-018-0900-5

**Published:** 2018-09-03

**Authors:** Michele Lanza, Michele Rinaldi, Ugo Antonello Gironi Carnevale, Silvio di Staso, Mario Bifani Sconocchia, Ciro Costagliola

**Affiliations:** 1 Multidisciplinary Department of Medical, Surgical and Dental Sciences, Università della Campania, Luigi Vanvitelli, Via de Crecchio 16, 80100 Naples, Italy; 2 Ophthalmology Unit, Department of Life, Health and Environmental Sciences, University of L’Aquila, L’Aquila, Italy; 3 Department of Medicine and Healthy Sciences, Università del Molise, Campobasso, Italy

**Keywords:** Innovative technology, Scheimpflug camera, Goldmann applanation tonometry, Corvis, naïve eyes, Healthy eyes, Rebound tonometry, ORA, No contact tonometry

## Abstract

**Background:**

To evaluate differences of intraocular pressure (IOP) measurements performed with Goldmann applanation tonometer (GAT), dynamic contour tonometer (DCT), rebound tonometry (RT), Ocular Response Analyzer (ORA) and Corvis ST (CST) in eyes screened for refractive surgery.

**Methods:**

One eye, only the right one, of 146 patients was included in this study. Each participant was submitted to a corneal analysis with Scheimpflug camera and IOP evaluation with GAT, DCT, RT, ORA and CST. Differences in IOP values obtained thanks to each instruments were compared and then correlations between these discrepancies and morphological features such as mean keratometry (MK) and central corneal thickness (CCT) provided by Pentacam were studied. Software used to run statistical evaluations was SPSS, version 18.0.

**Results:**

Study participants had a mean age of 33.1 ± 9.2 years old. IOP values observed in this study were 15.97 ± 2.47 mmHg (GAT), 17.55 ± 2.42 mmHg (DCT), 17.49 ± 2.08 mmHg (RT), 18.51 ± 2.59 mmHg (ORA) and 18.33 ± 2.31 mmHg (CST). The mean CCT was 560.23 ± 31.00 μm, and the mean MK was 43.33 ± 1.35 D. GAT provided significant lower values in comparison to all other devices. DCT and RT gave significantly lower intermediate IOP values than those measured with ORA and CST. All the IOP measures and the differences between devices were significantly correlated with CCT.

**Conclusions:**

According to our data, although our findings should be confirmed in further studies, GAT tonometer cannot be used interchangeably with DCT, RT, ORA and CST.

## Background

Intraocular pressure (IOP) evaluation is a crucial phase of a routine eye examination, particularly for glaucoma patients. Indeed, in these cases, elevated IOP is the only risk factor that physicians are able to modify [1]. This is the reason for the importance of the patient’s IOP value: it is a crucial element of glaucoma diagnosis and management [2].

Goldmann applanation tonometry (GAT) represents the “gold standard” method for IOP evaluation [3]. However, many factors may affect its precision. Among these, there are those related to the morphology of the eye, such as central corneal thickness (CCT) or corneal curvature, and those related to corneal biomechanical properties. CCT has been demonstrated to bias IOP measurements by GAT, inducing IOP underestimation in thin corneas and overestimation in thick ones [4]. Different formulas have been used to improve GAT precision, in an attempt to adjust IOP on the basis of CCT, but there is still not one capable of providing reliable and precise results [3, 5]. In order to overcome the problem, new tonometers have been developed. These have been designed to avoid the bias related not only to the corneal morphological properties and ocular surface, but also to corneal biomechanical properties, even though the real influence of the latter has not yet been completely established [6–12]. The most important goal of new devices to measure IOP is to provide accurate evaluations, free from the well-known limitations of GAT [13, 14]. Dynamic contour tonometry [6] (DCT Swiss Microtechnology AG, Port, Switzerland), rebound tonometry [9] (RT, Icare, Tiolat Oy, Helsinki, Finland), Ocular Response Analyzer [7] (ORA, Reichert, Buffalo, NY, USA) and Corvis ST [10] (CST, Oculus, Wetzlar, Germany) are devices capable of measuring IOP in different ways and they have been evaluated and compared to GAT in healthy subjects in this study.

Although studies on repeatability, reproducibility and comparisons among tonometers have already been published [12, 15–24], for the first time we provide a comparison among these 5 devices in naïve eyes and an analysis of differences related to corneal morphological parameters in a large population.

## Methods

In this prospective study 146 consecutive healthy subjects (62 females and 84 males), screened for refractive surgery, were included and evaluated. Every participant with any kind of illness (systemic or ocular) which could potentially affect the measurements of the parameters analyzed in the study were excluded, in order to have an unbiased statistical evaluation. Contact lens-wearing participants were asked to stop using them at least one week prior to examination. As protocol of the study, a routine eye examination was performed together with a Pentacam (Oculus, Wezlar, Germany) scan and IOP evaluations with GAT, DCT, RT, ORA and CST.

The Oculus Pentacam is a device which provides information in the form of maps and data regarding anterior and posterior corneal surface, depth of anterior chamber, corneal thickness and details about the lens [25]. Among the parameters it provides to measure anterior corneal power, Sim’K (MK) and CCT at pupil center were selected to be analyzed in this study.

DCT (Swiss MicrotechnologyAG, Port, Switzerland) is a contact tonometry that relies on the law of Blaise Pascal hydrostatic pressure [6].

RT is a contact tonometer that allows the measurement of the IOP thanks to a very small magnetized probe [9].

ORA is a non-contact device able to evaluate IOP by taking into account corneal biomechanical properties [7].

CST (Oculus, Wetzlar, Germany) is a recently introduced non-contact device that analyzes corneal deformation due to a constant air puff impulse. By measuring corneal deformation, this device provides some corneal biomechanical characteristics and IOP evaluations [10].

After Pentacam evaluation, ORA, CST, RT, DCT and GAT measurements were taken in this sequence in order to obtain the most accurate IOP evaluations possible, reducing errors due to potential corneal deformation. Thus, the more “invasive” devices were used at the end. Each measurement was performed 10 min after the previous one, and three consecutive IOP measurements for each instrument were collected and averaged; the mean value was utilized for statistical evaluations.

All IOP evaluations were completed between 2:00 pm and 4:00 pm. Eyes with corneal anomalies, such as corneal thickness increase, corneal disepithelization or corneal curvature alterations, documented at the end of IOP measurements, were also excluded from the study. Each device was associated with an operator who performed the evaluation, unaware of the results obtained by the other physicians with the other equipments.

Study participants signed an informed consent form before starting examinations; this study followed the ethical standards of the 1964 Declaration of Helsinki and approved by the local clinical research ethics committee.

### Statistical analysis

Even though both eyes of the participants were evaluated, only the right eye was selected to be used in the statistical analysis in order to avoid any potential intra-subject effect. Normality of distribution of the study population was analyzed with the Kolmogorov-Smirnov test. In this study analysis of differences and correlations of data not reaching the normality standards was performed using non-parametric tests. Particularly, a Friedman test, as a non-parametric alternative to ANOVA, was performed, followed by a post-hoc Wilcoxon signed rank test to evaluate comparisons among values obtained from different instruments. Moreover, considering that we did 10 pairwise comparisons, *p*-value of each comparison was adjusted using the Bonferroni method (pa = px10). Furthermore, the correlations among CCT and MK vs IOP values obtained with tested devices were evaluated using non-parametric (Spearman) tests. After analysis of the study population and evaluation of the error of the tested devices, the level of significance was set at *p* < 0.05 for all statistical tests. Statistical evaluations were performed using SPSS software (IBM Corp. Armonk, New York) version 18.0.

## Results

**Fig. 1:**
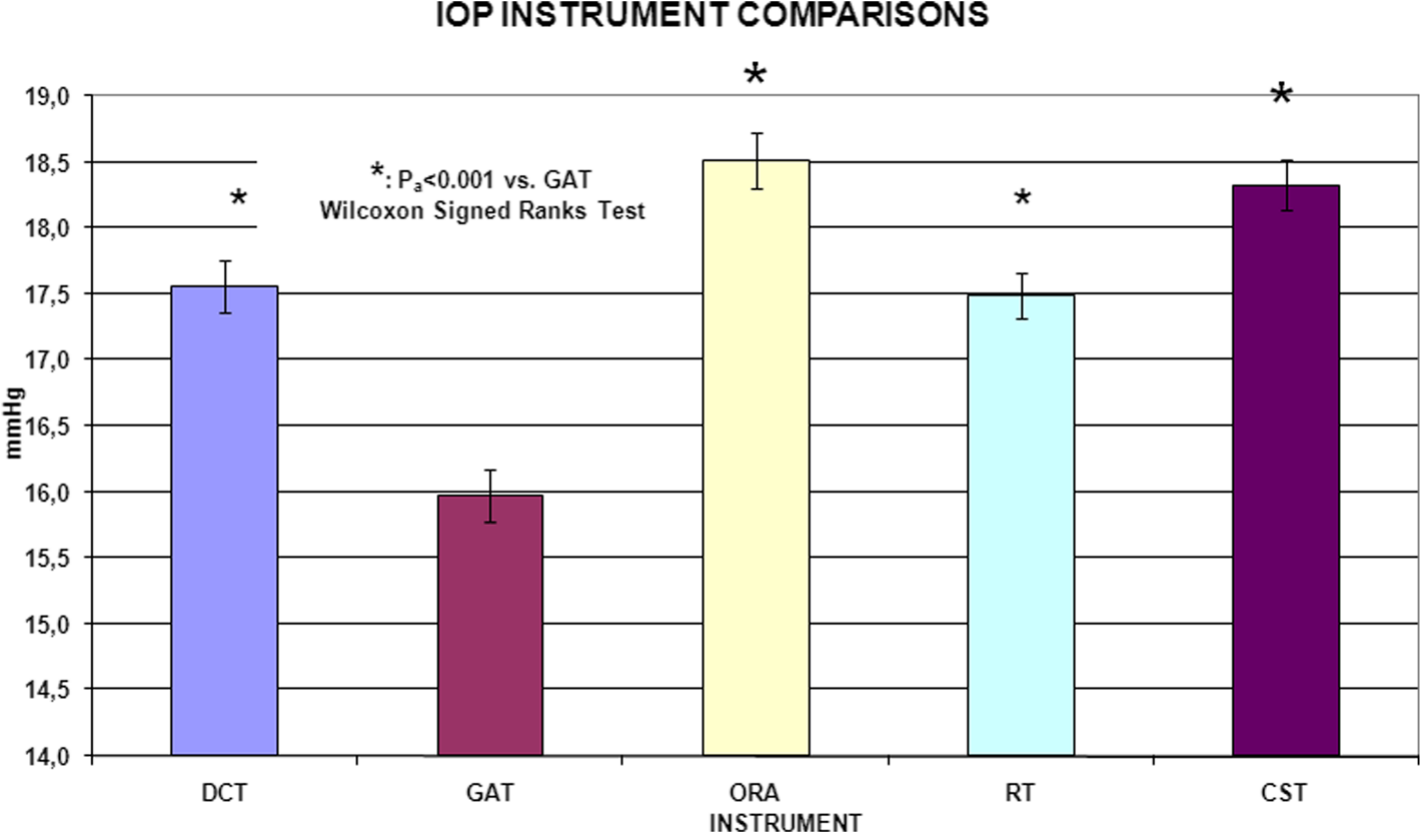
Range (expressed as mean ± standard deviation) of intraocular pressure measurements in healthy participants observed using Ocular Response Analyzer, Goldmann tonometer, Dynamic Contour Tonometry, Rebound tonometer and Corvis tonometer; statistical differences expressed as * when *p*_a_ < 0.001

**Fig. 2:**
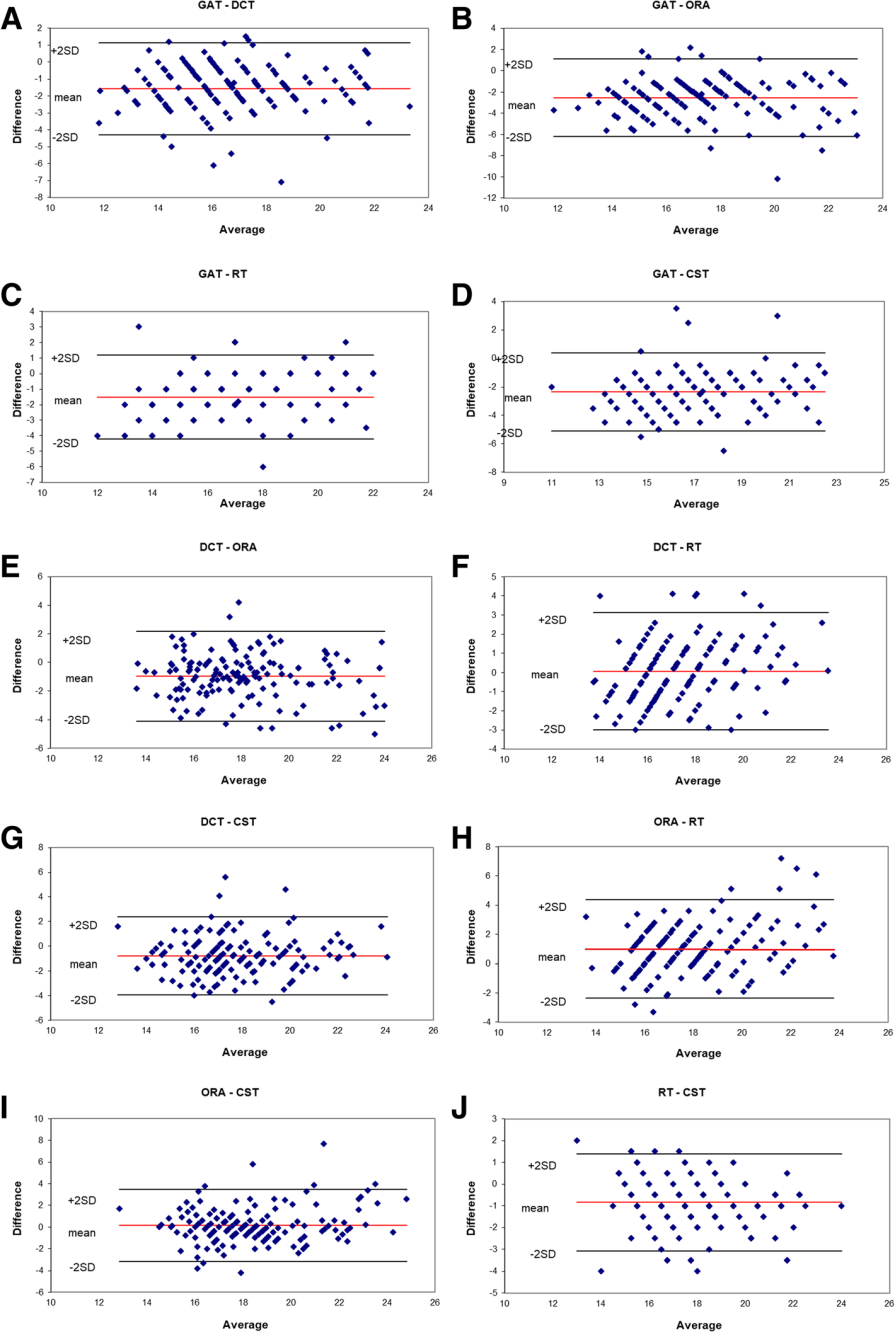
Bland & Altman plots. **a** Goldman vs Dynamic Contour Tonometry; **b** Goldman vs Ocular Response Analyser; **c** Goldman vs Rebound tonometry; **d** Goldman vs Corvis tonometry; **e** Dynamic Contour Tonometry vs Ocular Response Analyser; **f** Dynamic Contour Tonometry vs rebound tonometry; **g** Dynamic Contour Tonometry vs Corvis; **h** Ocular Response Analyser vs rebound tonometry; **i** Ocular Response Analyser vs Corvis tonometry; **j** Rebound tonometry vs Corvis

The participants in the study were aged between 19 and 55 years old (mean: 33.1 ± 9.22 years) with a mean refraction, calculated as spherical equivalent (SE), of − 4.65 ± 2.03 D (ranging from − 10.25 D to 0 D). In particular, there were 47 eyes with myopia, 98 ones with myopic astigmatism and only one with mixed astigmatism. Details of demographical and morphological parameters of the subjects included in this study are shown in Table 1. Table 2 and Fig. 1 show the IOP values obtained with the tested instruments. A preliminary analysis confirmed significant differences among the values of different instruments (F_R_: 288.71, d.f. 4, *p* < 0.001; Friedman test). Overall, GAT values showed significantly lower values in comparison with those obtained from other devices. IOP measurements obtained by the tested tonometers were plotted by the means of Bland et Altman plots (Fig. 2). ORA showed the largest IOP overestimation compared to GAT (+ 2.54 mmHg; *p*_a_ < 0.001, Fig. 2b). Differences between CST and GAT (+ 2.35 mmHg; *p*_a_ < 0.001, Fig. 2d), between DCT and GAT (+ 1.58 mmHg; *p*_a_ < 0.001, Fig. 2a) and between RT and GAT (+ 1.52 mmHg; *p*_a_ < 0.001 Fig. 2c) were also significant.

**Table 1:** Clinical characteristics of patients included in the study

Characteristic	Mean ± SD	Range
Age (year)	33.10 ± 9.22	from 19 to 55
Spherical equivalent (D)	−4.65 ± 2.03	from − 10.25 to 0
Corneal curvature (D)	43.33 ± 1.35	from 40.1 to 46.6
Corneal pachymetry at pupil center (μm)	560.23 ± 31.00	from 500 to 665

*SD* standard deviation

**Table 2:** IOP differences between tested tonometers (mmHg, Wilcoxon test)

	Mean (mmHg)	*p*_a_ value
DCT - GAT	+ 1.580	0.0001
ORA - DCT	+ 0.958	0.0001
DCT - RT	+ 0.064	0.62
CST - DCT	+ 0.775	0.0001
ORA - GAT	+ 2.538	0.0001
RT - GAT	+ 1.516	0.0001
CST - GAT	+ 2.355	0.0001
ORA - RT	+ 1.022	0.0001
ORA - CST	+ 0.183	0.194
CST - RT	+ 0.839	0.0001

Legend: Legend: Mean IOP difference between tested devices (pa: Bonferroni adjusted *p*-value); Corvis ST (CST), Ocular Response Analyzer (ORA), rebound tonometry (RT), dynamic contour tonometer (DCT) and Goldmann applanation tonometer (GAT)

Table 3 shows the correlations and their significances between IOP values provided by the tested devices and other parameters such as age, spherical equivalent, MK and CCT.

**Table 3:** Correlations between IOP, as obtained from tested tonometers, and age, spherical equivalent (SE), corneal curvature (MK) and central corneal thickness (CCT)

Correlations					
		AGE	SE	MK	CCT
DCT	Spearman’s rho	−0.117	0.025	−0.067	0.470
	Sig. (2-tailed)	0.159	0.762	0.422	**0.0001**
	N	146	146	146	146
GAT	Spearman’s rho	−0.143	0.102	− 0.043	0.625
	Sig. (2-tailed)	0.085	0.221	0.608	**0.0001**
	N	146	146	146	146
ORA	Spearman’s rho	−0.089	−0.029	− 0.096	0.413
	Sig. (2-tailed)	0.283	0.732	0.251	**0.0001**
	N	146	146	146	146
RT	Spearman’s rho	−0.114	0.072	−0.098	0.519
	Sig. (2-tailed)	0.172	0.389	0.240	**0.0001**
	N	146	146	146	146
CST	Spearman’s rho	−0.104	0.040	−0.201	0.522
	Sig. (2-tailed)	0.210	0.633	**0.015**	**0.0001**
	N	146	146	146	146

Legend: Corvis ST (CST), Ocular Response Analyzer (ORA), rebound tonometry (RT), dynamic contour tonometer (DCT) and Goldmann applanation tonometer (GAT). Highlighted values are the correlations resulted to be significant

## Discussion

Lowering IOP is the most important aid that physicians can offer to block or reduce glaucoma progression [1], thus a precise and reliable estimation of this is extremely important. Because glaucoma is a chronic degenerative disease and IOP values must be recorded for a patient’s whole life, it is extremely important that a device able to measure IOP without any bias should be available for eye doctors. It is well known that the current gold standard, GAT, does not always provide very precise measurements but new IOP measuring devices haven’t shown uniform accuracy, according to previously published papers [12, 21–29].

Each tonometer tested in this study evaluates IOP with different working principles; three are contact tonometers (GAT, DCT and RT) whereas two do not require any contact (ORA and CST). Even though each one resulted in some way as being dependent on CCT, CST seems to be influenced by MK too (Table 3).

DCT, as already reported by Schneider et al. [26], is more suitable for measuring IOP in cooperative patients with sufficient bilateral ocular fixation, however, some patients are not sufficiently compliant. ORA and CST, being non-contact tonometers, are less invasive for patients and thus they can also be used particularly in clinical situations where it is better to avoid direct corneal contact (corneal infections, recent corneal surgery). RT operates through very soft and well-tolerated contact between the probe and the cornea; however, its results could be affected by tear film more than the other tested devices [24].

Each tonometer alternative to GAT tested in this study provided significantly higher IOP values, probably because of their working principles. It is not possible to establish which one is the most reliable, since they should be compared with real IOP measurements obtained only by intraocular probe.

The results observed herein agree with most of the previously published papers analyzing the differences among tested devices on healthy subjects [11, 12, 17, 19–22, 24, 26–29]. In this study, a strict measurement order going from the less supposedly “invasive” device for cornea to the one considered to be the most “invasive”, was followed, whereas in other papers a random order was often adopted [10, 29]. The order of IOP measurements chosen in this study may introduce some other kind of bias, due to the fixed sequence and to the number of devices tested. In this paper differences between ORA and CST are not statistically significant whereas a statistical difference of 1.25 mmHg was observed in a previous paper by the same group of authors [11]. The explanations for this data could be related to different reasons: the group of healthy subjects analyzed in this paper is larger (146 vs 76) compared to the previous one, no participants of the other study contributed to this one, CST software has changed over time and this could be one of the reasons for these different observed results. It is important to underline that the refractive defect of the participants in the study is mostly myopic; this need to be considered in comparison with other papers.

Another limit of this study could be related to the long time (about 40 min) which was needed to perform all the IOP measurements on the participants, because an IOP fluctuation occurs during this time. It is important to underline that, in order to minimize this kind of bias, every IOP evaluation was performed between 2:00 pm and 4:00 pm and during this time IOP fluctuation has been demonstrated to be about 0.5 mmHg whereas it is much higher in the early hours of the morning, about 2 mmHg from 7:00 a.m. to 9:00 a.m. [30, 31].

Comparing devices capable of measuring IOP is always a complex procedure, also because physicians are currently using instruments that perform indirect evaluations. There are few studies comparing the IOP obtained with these devices with IOP recorded with invasive intra-cameral manometry of the anterior chamber [32]. Even though both healthy subjects and glaucoma patients are evaluated in this study, the authors found an overestimation of DCT compared to GAT and that manometric IOP values are lower than GAT ones [32]. This could be useful to take in account the differences of the tonometers evaluated in this study.

## Conclusions

Results observed in the current study suggest that each device evaluated provides an overestimation of IOP compared to GAT. This is not claiming that one of them is more accurate than the others but, according to this data, it is still not possible to obtain IOP values which are not influenced by corneal morphological parameters and if one of these new tonometers is adopted as the gold standard in the future, new IOP limits need to be set when evaluating the risk of the development of glaucoma.
